# Role of mtDNA Haplogroups in the Prevalence of Knee Osteoarthritis in a Southern Chinese Population

**DOI:** 10.3390/ijms15022646

**Published:** 2014-02-14

**Authors:** Hezhi Fang, Xinwei Liu, Lijun Shen, Fengjie Li, Yihong Liu, Hongbo Chi, Huikai Miao, Jianxin Lu, Yidong Bai

**Affiliations:** 1 Key Laboratory of Laboratory Medicine, Ministry of Education, Zhejiang Provincial Key Laboratory of Medical Genetics, Wenzhou Medical University, Wenzhou 325035, Zhejiang, China; E-Mails: hezhifang990909@gmail.com (H.F.); ysslj23@163.com (L.S.); lifengjie653824@163.com (F.J.); liuyihongloe@163.com (Y.L.); sxnydx.sk073.chb@163.com (H.C.); miaohuikai1989@163.com (H.M.); 2 Department of Orthopedics and Rescue Center of Severe Wound and Trauma of Chinese PLA, General Hospital of Shenyang Military Area Command of Chinese PLA, Shenyang 110016, Liaoning, China; E-Mail: deformitya@126.com; 3 Department of Cellular and Structural Biology, University of Texas Health Science Center at San Antonio, San Antonio, TX 78229, USA

**Keywords:** mitochondria, haplogroup, osteoarthritis, copy number

## Abstract

Mitochondrial DNA (mtDNA) has been implicated in various human degenerative diseases. However, the role of mtDNA in Osteoarthritis (OA) is less known. To investigate whether mtDNA haplogroups contribute to the prevalence of knee OA, we have carried out a comprehensive case-control study on 187 knee OA patients and 420 geographically matched controls in southern China. OA patients were classified on the Kellgren/Lawrence scale from two to four for the disease severity study and the data were analyzed by adjusting for age and sex. We found that patients with haplogroup G (*OR* = 3.834; 95% CI 1.139, 12.908; *p* = 0.03) and T16362C (*OR* = 1.715; 95% CI 1.174, 2.506; *p* = 0.005) exhibited an increased risk of OA occurrence. Furthermore, patients carrying haplogroup G had a higher severity progression of knee OA (*OR* = 10.870; 95% CI 1.307, 90.909; *p* = 0.007). On the other hand, people with haplogroup B/B4 (*OR* = 0.503; 95% CI 0.283, 0.893; *p* = 0.019)/(*OR* = 0.483; 95% CI 0.245, 0.954; *p* = 0.036) were less susceptible for OA occurrence. Interestingly, we found OA patients also exhibited a general increase in mtDNA content. Our study indicates that the mtDNA haplogroup plays a role in modulating OA development.

## 1. Introduction

Osteoarthritis (OA) is an age related degenerative disease characterized by the degradation of articular cartilage, caused by dysfunctional chondrocytes. Risk factors for OA include gender, occupation, smoking, as well as nuclear genetic background such as mutations in genes encoding IGF-1, ER alpha, cartilage matrix protein, aggrecanase-2 and Bradykinin B2 [1–3]. The chondrocyte is the only cell type in cartilage, and its dysfunction is the main hallmark of OA [4]. Recent studies have indicated a role for mitochondria in the development of OA. OA cells were shown to have decreased expression of respiratory complexes II and III [5]. Moreover, inhibition of complex I and IV activity increased mitochondrial related chondrocyte apoptosis [5]. The underlying mechanisms mediating the mitochondrial dysfunction in OA have been suggested as increased chondrocyte oxidative stress and apoptosis, decreased chondrocyte biosynthesis, upregulated chondrocyte inflammation and matrix catabolism, and accelerated cartilage matrix calcification [4].

It is estimated that mitochondria generate more than 90% of cellular ATP. Human mitochondria contain over 1000 proteins, and 13 of them are encoded by mtDNA. mtDNA mutations have been reported to account for many human diseases [6,7], and many mtDNA variants that occurred during selective events over the long period of human evolution were considered as sites of polymorphism [8]. A specific mtDNA haplogroup/haplotype represents a set of single nucleotide polymorphisms (SNPs), which are genetically and statistically associated. There are many independent studies shown that genetic variations in mtDNA can modulate mitochondrial functions such as calcium dynamics, respiratory complex activity, and mtDNA transcription and replication [9–11]. In particular, multiple case-control studies have revealed that mtDNA haplogroups are related to various complex diseases such as metabolic syndromes [12,13], neurodegenerative diseases [14,15], infectious diseases [16,17], cancer [18,19], and aging [20].

The prevalence of OA in China is comparable with that in Europe. The number of knee osteoarthritis cases in the urban elderly population from Southern China is about 13%, and is higher in rural areas [21–23]. Recent studies have demonstrated that mtDNA haplogroups play a role in the prevalence of both hip and knee OA in Spanish populations. However, the reported OA related haplogroup J, was only found in Europe, while the major mtDNA haplogroups in China are A, B, CZ, D, M7, M8 and F. Haplogroup F and B were more frequently distributed in Southern China than in Northern China [24]. To further assess the possible contribution of mtDNA haplogroups to the prevalence of OA and the potential mechanisms that underlie the relationships between OA and mtDNA haplogroups, we conducted a study between knee OA and mtDNA haplogroups in a Southern Chinese population.

## 2. Results and Discussion

### 2.1. Result

#### 2.1.1. Age and Gender in the Occurrence of OA

**Figure 1.**
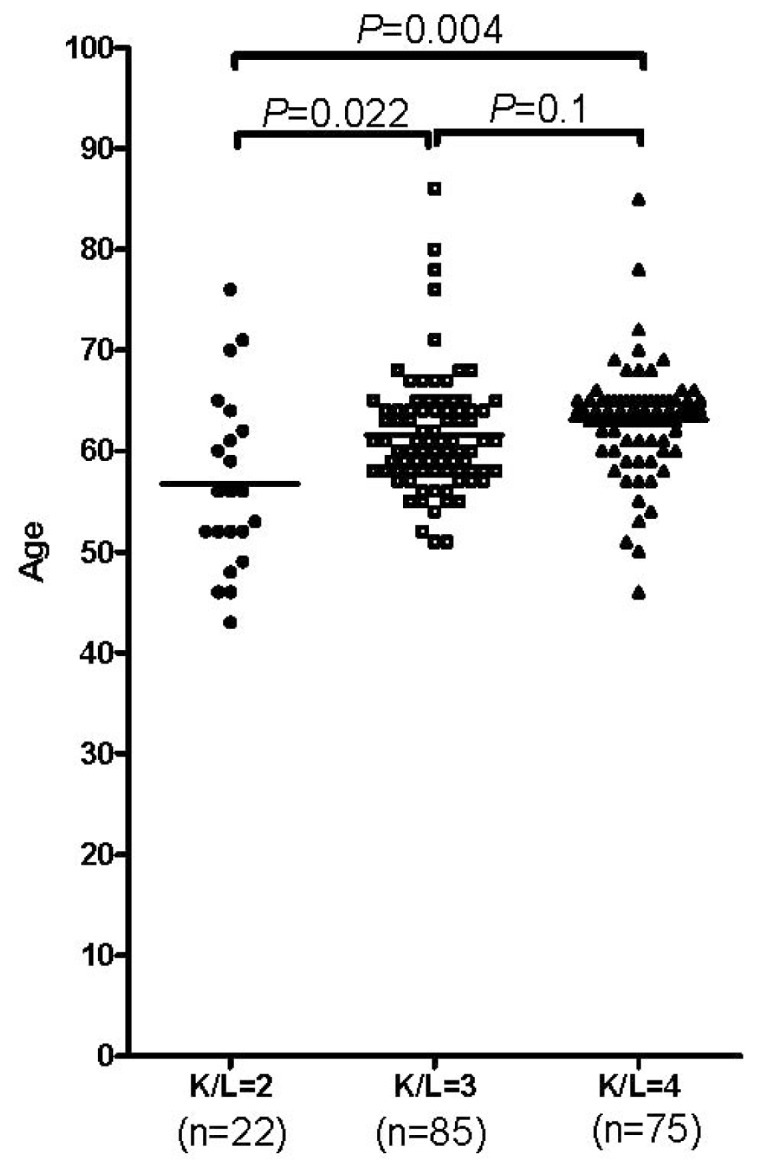
Influence of age in the occurrence of OA. *p* values were estimated by “One-Way ANOVA”; K/L, Kellgren/Lawrence.

We first analyzed two clinical characteristics, age and gender, in the OA occurrence. As shown in Table 1, the frequency of women (72.19%) was significantly higher in patients with OA than it was (56.42%) in the control group (*OR* = 0.497; 95% CI 0.342, 0.722; *p* < 0.001) which indicates that women have a higher chance to develop OA than men. The result remained significant (*OR* = 0.537; 95% CI 0.362, 0.796; *p* = 0.002) after an age-adjustment of the data in a binary regression analysis. In addition, Figure 1 showed the close relationship between age and OA development. Though the age distribution between K/L 3 (mean age 61.60) and K/L 4 (mean age 63.60) group has no difference (*p* = 0.1), patients in both K/L 3 and K/L 4 group were much older than those patients in K/L 2 (mean age 56.77) group (*p* = 0.022 for K/L 2 and 3; *p* = 0.004 for K/L 2 and 4).

#### 2.1.2. mtDNA Haplogroup and SNPs Analysis in OA Patients

The frequency of haplogroup G was significantly higher in OA patients than in the controls (*OR* = 3.084; 95% CI 1.055, 9.017; *p* = 0.031) (Table 2). This significant correlation remained after adjusting with age and gender (*OR* = 3.834; 95% CI 1.139, 12.908; *p* = 0.03). However, the frequency of haplogroup R showed lower frequency in OA patients when compared with controls (*OR* = 0.687; 95% CI 0.473, 0.998; *p* = 0.048). As shown in Table 2, we found the effect of haplogroup R was contributed by F1a and B, and sub-haplogoup B4, which directly belongs to B. After adjusting for age and gender, only haplogroup B (*OR* = 0.503; 95% CI 0.283, 0.893; *p* = 0.019) and its sub-haplogroup B4 (*OR* = 0.483; 95% CI 0.245, 0.954; *p* = 0.036) exhibited a significantly lower occurrence in OA patients. No other macro or sub-haplogroups in this study were found associated with OA before and after logistic regression analysis (Table 2).

Consistent with the result that haplogroup B and B4 have a protective effect on OA occurrence, analysis of the contribution of individual SNPs in OA occurrence showed that both SNP T16140C and T16217C, which mostly determined haplogroup B and B4 in the Chinese Han population, were less represented in OA patients (Table 3). Following age and gender adjustment in a binary regression analysis, the result is still significant for T16140C (*OR* = 0.370; 95% CI 0.142, 0.958; *p* = 0.041) and T16217C (*OR* = 0.493; 95% CI 0.249, 0.977; *p* = 0.043). For the analysis of the rest of the mtSNPs listed in Table 3, T16362C was found associated with increased OA incidence after adjusted by age and gender (*OR* = 1.715; 95% CI 1.174, 2.506; *p* = 0.005).

To further understand the role of mtDNA on OA development, or associations of mitochondrial haplogroup, or mtDNA SNPs with the severity of OA, patients were divided into a K/L 2–3 group and a K/L 4 group for evaluation (Tables 4 and 5). The results showed a significantly higher frequency of haplogroup G in the high severity group (K/L 4) (*OR* = 10.870; 95% CI 1.307, 90.909; *p* = 0.007). For haplogroup B (*OR* = 0.812; 95% CI 0.304, 2.169; *p* = 0.677) and its sub-haplogroup B4 (*OR* = 0.880; 95% CI 0.277, 2.801; *p* = 0.829), no significant results were found. In addition, though haplogroup D4 and its sub-haplogroup D4a, haplogroup N9 and its sub-haplogroup N9a are neither risk factors nor protective factors of OA occurrence, patients with haplogroup D4/D4a usually develop higher severity OA while those patients with haplogroup N9/N9a tend to develop lower severity OA (Table 4). Despite the lack of significant association between mtDNA SNPs and severity detected, the mtDNA risk SNP T16362C showed a trend towards a higher severity (Table 5).

#### 2.1.3. mtDNA Copy Number Alternation in OA Patients

**Figure 2.**
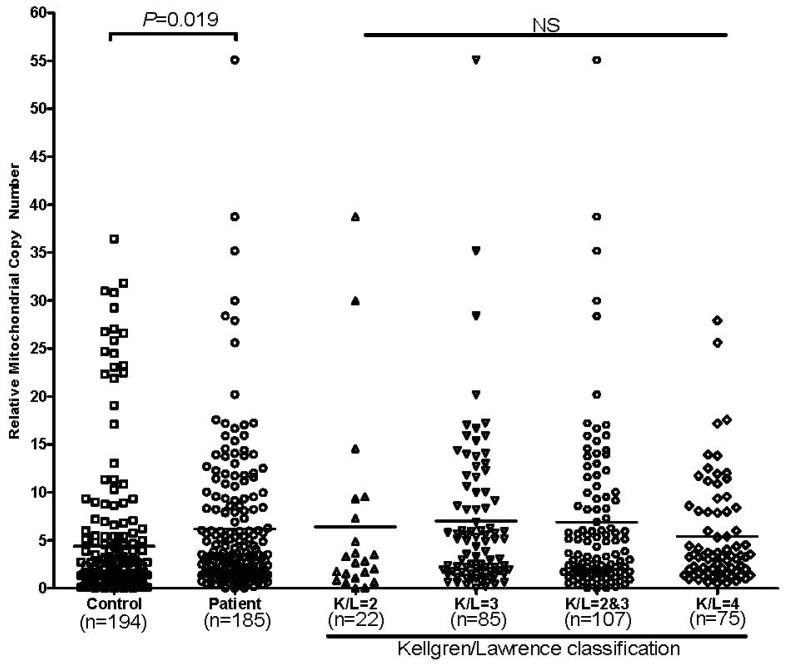
Effect of mtDNA copy number in OA occurrence and OA development. For patients and controls comparison, *p* values were estimated by “Student’s unpaired *t* test”; for K/L group comparison *p* values were estimated by “One-Way ANOVA”; K/L, Kellgren/Lawrence.

Previous reports showed mtDNA level was modulated by mtDNA haplogroup [9]. To understand whether mtDNA copy number alterations affected OA, we measured mtDNA copy number in 185 patients and 194 random selected controls. As shown in Figure 2, OA patients have increased mtDNA copy number (mean ± SD, 6.21 ± 7.478) compared with controls (mean ± SD, 4.4 ± 7.459) (*p* = 0.019), the result remains significant after adjust by age and gender (*p* = 0.033). Further analysis of the mtDNA copy number in OA patient with different K/L grade showed no difference.

**Figure 3.**
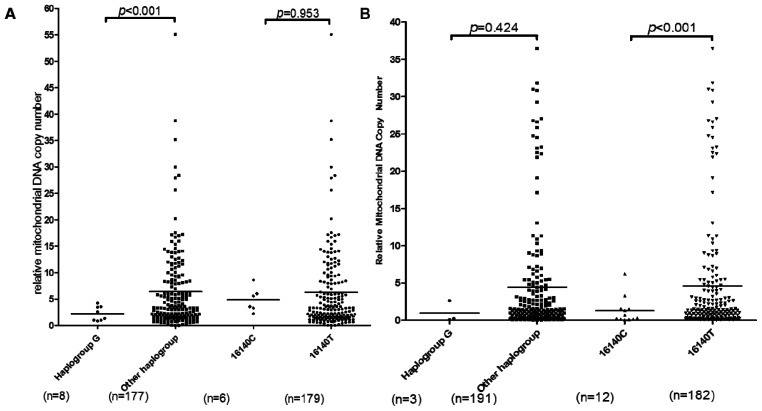
Mitochondrial DNA haplogroup/mtSNPs affect OA by changes in compensatory upregulation ability of mtDNA content. (**A**) mtDNA content in OA patient; (**B**) mtDNA content in controls; *p* values were estimated by “Student’s unpaired *t* test”.

To test the correlations between mtDNA background and mtDNA copy number in OA, OA patients and controls were regrouped by OA-specific mtDNA haplogroup/mtSNPs, then reanalyzed by the mtDNA content alteration in those groups. For haplogroup G, mtDNA content was lower in haplogroup G patients (mean ± SD, 2.233 ± 1.36) compared with non-haplogroup G patients (mean ± SD, 6.385 ± 7.69) (*p* < 0.001) while there was no difference between haplogroup G (mean ± SD, 0.97 ± 1.43) and non-haplogroup G (mean ± SD, 4.45 ± 7.5) in controls (*p* = 0.424) (Figure 3A,B). These results indicate that haplogroup G was less likely to associate with the upregulation of mitochondrial function by increasing mtDNA copy number than other haplogroups in OA development. For mtSNP16140, mtDNA content was significant higher in 16140T controls (mean ± SD, 4.604 ± 7.64) compared with 16140C controls (mean ± SD, 1.279 ± 1.82) (*p* < 0.001) while there is no difference between 16140T (mean ± SD, 6.250 ± 7.58) and 16140C (mean ± SD, 4.859 ± 2.32) in OA patients (*p* < 0.953) (Figure 3A,B). It suggests 16140C is more associated with the likelihood to maintain mitochondrial function by increasing mtDNA copy number than 16140T in OA development. But again, due to the lower prevalence of haplogroup G in Southern China, we used a small sample size in the mtDNA copy number related OA study. For other OA specific mtDNA haplogroup/mtSNPs, no copy number differences were found in either patients or controls.

### 2.2. Discussion

The most common type of arthritis, Osteoarthritis, affects patient movement due to its painful phenotype and can have either genetic or environmental risk factors [25,26]. In this study, we recruited 187 knee OA patients and 420 controls to investigate whether human mitochondrial genetics influence the susceptibility and development of OA in Chinese populations.

Consistent with a previous clinical epidemiological study on OA [27], we found women to be more susceptible to developing OA than men. Additionally, elder age groups developed more severe of OA, helping confirm that OA is an age related degenerative disease.

Previous case control studies stated that haplogroup J showed a significant decrease in risk for both hip OA and knee OA in Spanish populations [28,29]. Specifically, haplogroup J had a lower severity of development in knee OA [29]. However, those results conflicted with another recently published study using patients from the UK, which claimed that evidence linking OA to mtDNA has not been well established [30]. However, the contribution of mtDNA to OA can differ depending upon ethnic group and environmental factors. In the current study, we focused on a Southern Chinese population. The major results obtained from our study showed that haplogroup G and mtSNPs T16362C increased the risk of OA. Additionally, haplogroup B, which was contributed by sub-haplogroup B4, and their haplogroup diagnostic mtSNPs T16140C and T16217C decrease the susceptibility of OA.

This is the first time we provide a link between haplogroup G and the risk of knee OA. In this study, we also found patients carrying haplogroup G having a higher susceptibility to develop severely progressing OA. Since mitochondrial defects are one of the major causes of aging related degenerative diseases such as OA [4], it is conceivable that the finding in our study, that OA patients have upregulated mtDNA copy number, is a compensatory mechanism for mitochondrial defects. For haplogroup G, a previous report showed haplogroup G can increase the mtDNA deletion and mitochondrial ROS generation thereby associated with the increased risk of lung cancer [31]. In our study, we did not see such compensatory effects in the peripheral blood cells containing haplogroup G in OA patients, meaning cells like chondrocytes containing mtDNA haplogroup G background are prone to be injured due to the fact that their mitochondria are vulnerable under oxidative stress. Similarly, chondrocytes with 16140C mtDNA genetic background can protect chondrocytes from oxidative stress induced damage and apoptosis by increasing the mitochondrial function with up regulated mtDNA copy number. Subsequently, we found mtDNA haplogroup B/B4, which was reported as a protected haplogroup on the occurrence of biliary atresia and oral lichen planus, has protective effect on OA occurrence in our study [32,33]. Further functional studies confirmed that haplogroup B/B4 have increased mitochondrial membrane potential and mtDNA copy number, as well as decreased ROS levels and cell apoptosis under stress [33]. All these functional alternations support that haplogroup B/B4 exhibited important protective effects on chondrocytes. In addition, both of ours and previous studies claimed haplogroup B/B4 and haplgroup J, which are the sub-haplogroups of macro haplogroup R, are protective mtDNA haplogroups in Chinese and Spanish OA patients respectively [34], but the other subhaplogroup R9 and R11 also from macro haplgroup R do not have such protective effect. It is indicated that different mtDNA variations rather than the shared mtDNA variations from macro haplogroup R determine the protective effect of haplogroup B/B4 and haplgroup J on OA.

T16362C is the only non-haplogroup related mtSNP to increase OA incidence in our study. T16362C itself and the combination of T16362C and G11778A have been associated with breast cancer in Poland and older age onset of Leber’s hereditary optic neuropathy (LHON) in Japan respectively [35,36]. Though there has not yet been a complementary functional study available for T16362C, both of these previous studies and our work show that T16362C is a risk factor for human degenerative diseases. Finally, based on the results obtained from this study, a larger sample size is required to confirm the effect and the potential role of mtDNA haplogroup on OA.

## 3. Experimental Section

### 3.1. Subjects

A total of 187 unrelated patients (mean ± SD age 61.59 ± 6.283 years, range 43–86) with knee OA were recruited at the Changhai Hospital of Second Military Medical University from May 2011 to June 2013. Patients (135 women and 52 men) were diagnosed with knee OA according to the American College of Rheumatology (ACR) criteria. Based on the radiographs of the knee OA patients, the severity of OA was determined according to the Kellgren/Lawrence (K/L) grade classification from grade one to four and only patients with K/L grade 2 or higher were included in this study. A total of 420 geographically matched asymptomatic control subjects (mean ± SD age 55.32 ± 8.98 years, range 27–84, 237 women and 183 men) who did not meet the criteria for knee OA and other known degenerative diseases including cancer, diabetes and hypertension, which could be associated with mitochondrial defects, were also recruited at Changhai Hospital of the Second Military Medical University. Informed consent was obtained from all subjects under protocols approved by the Ethical Committee of the Second Military Medical University.

### 3.2. mtDNA Sequencing and Genotyping

Genomic DNA was extracted from peripheral blood using a SDS lysis protocol as described [37]. Two pairs of primers were designed to amplify the mtDNA fragments containing major diagnostic SNPs of the Asian mtDNA haplogroup. The primers sequences were as follows, L15975F: 5′-CTCCACCATTAGCACCCAAAGC-3′, H794R: 5′-AGGCTAAGCGTTTTGAGCT′G-3′ and L9967F: 5′-TCTCCATCTATTGATGAGGGTCT-3′, H10858R: 5′-AATTAGGCTGTGGGTGGTTG-3′. An additional sequencing primer, 299F GGTGGAAATTTTTTGTTATG, was designed to sequence the potential poly C gap at mt16184 to mt16193. The PCR was performed using a PCR Amplification Kit (TaKaRa, Tokyo, Japan) on a Thermal Cycler 170-9703 PCR machine (Bio-Rad, Hercules, CA, USA). The PCR amplification procedures were as follows: pre-denaturation at 95 ºC for five minutes, followed by 35 cycles of (94 ºC for 30 s, 57 ºC for 30 s, 72 ºC for 40 s), and with a final extension at 72 ºC for four minutes. PCR products were purified using MiniBEST DNA Fragment Purification Kit Ver.3.0 (TaKaRa, Japan).

SNPs of each subject were obtained by comparing sequences with the revised Cambridge Reference Sequence (rCRS) using CodonCode Aligner 3.0.1 (CodonCode Corporation, Centerville, VA, USA). A specific mtDNA haplogroup was assigned to each subject by comparing the target SNPs from the d-loop, ND3 and ND4L with the diagnostic SNPs of the constructed East Asian mtDNA haplogroup tree [24,38].

### 3.3. mtDNA Copy Number Evaluation

A ratio of mtDNA *versus* nDNA was generated to represent the relative amount of mtDNA copy number. The Real-Time PCR reactions were performed on a Stepone Real-Time PCR system (Applied Biosystems, Foster City, CA, USA) using SYBR^®^ Green qPCR Mastermix (TaKaRa, Tokyo, Japan). The detailed human primers are listed as previous reported [39]: mtDNA (Human- tRNA leucine 1, Transcription terminator and 5S-like sequence): forward, 5′-CACCCAAGAACAGGGTTTGT-3′; reverse, 5′-TGGCCATGGGTATGTTGTTAA-3′. nDNA (18s ribosomal DNA): forward, 5′-TAGAGGGACAAGTGGCGTTC-3′; reverse, 5′-CGCTGAGCCAGTCAGTGT-3′. Two independent reactions were performed for mtDNA and nDNA of each sample. The PCR amplification procedures were as followers: pre-denaturation at 95 ºC for five minutes, followed by 40 cycles of (94 ºC for 30 s, 58 ºC for 30 s, 72 ºC for 60 s). The mtDNA copy number measured by mtDNA primers were normalized by nDNA copy number. PCR efficiencies of these primers were tested between 90% and 110%.

### 3.4. Statistical Analysis

All statistical analyses were performed using SPSS software (version 13.0) (IBM, Armonk, NY, USA). A null hypothesis was rejected when *p* < 0.05. The relationship between the prevalence of haplogroups or SNPs and OA were evaluated by using the Pearson chi-square test. Binary logistic regression analysis was used to test the contribution of haplogroup, gender and age to OA.

## 4. Conclusions

In conclusion, we found that haplogroup G and T16362C exhibit an increased risk of OA occurrence, and that haplogroup B, its sub-haplogroup B4, and their diagnostic mtSNPs T16140C and T16217C are protecting factors for OA occurrence. Our study is the first to report that mtDNA copy number was upregulated in OA patients. We are also the first to report that the genetic background of mtDNA can modulate OA incidence by affecting upregulation of mtDNA copy number in OA patients. However, more studies are still needed to better understand the role of mtDNA genetic background in OA.

## Figures and Tables

**Table 1.** Influence of gender in the occurrence of OA.

Gender	Patient (*n*)	Control (*n*)	*OR* (95% CI)	*p* value
Male	52	183	0.497(0.342, 0.722)	*p* < 0.001 *
Female	135	237		

*p* values were estimated by “chi-square test”;

(*) indicated statistical significant (*p* < 0.001);

OR indicates odds ratio; 95% CI, 95% confidence interval.

**Table 2.** Effect of mitochondrial DNA haplogroup in patients with knee OA.

Haplogroup	Patient (*n* = 187)	Control (*n* = 420)	*OR* (95% CI)	*p* value
**M**	103 (55.1)	211 (50.2)	1.215 (0.859, 1.716)	0.270
**D**	40 (21.2)	90 (21.4)	0.998 (0.655, 1.519)	0.992
**D4**	17 (9.1)	57 (13.6)	0.637 (0.360, 1.128)	0.119
**D4a**	4 (2.1)	18 (4.3)	0.488 (0.163. 1.463)	0.191
**D5**	21 (11.2)	31 (7.4)	1.587 (0.886, 2.844)	0.118
**D5a**	5 (2.7)	8 (1.9)	1.415 (0.457, 4.383)	0.546
**M8**	21 (11.2)	39 (9.3)	1.415 (0.457, 4.383)	0.546
**M8a**	7 (3.7)	10 (2.38)	1.594 (0.597, 4.2550)	0.348
**CZ**	14 (7.5)	28 (6.67)	1.136 (0.584, 2.211)	0.708
**M7**	8 (4.3)	32 (7.6)	0.542 (0.245, 1.2)	0.126
**M7b**	3 (1.6)	20 (4.8)	0.326 (0.096, 1.111)	0.06
**M7c**	4 (2.1)	8 (1.9)	1.114 (0.331, 3.744)	0.862
**G**	8 (4.3)	6 (1.4)	3.084 (1.055, 9.017)	0.031 *
**M9**	4 (2.1)	4 (1.0)	1.180 (0.291, 4.7860)	0.819
**M10**	5 (2.6)	8 (1.9)	1.415 (0.457, 4.384)	0.546
**N**	84 (44.9)	209 (49.8)	1.215 (0.859, 1.716)	0.270
**A**	18 (9.6)	35 (8.33)	1.171 (0.645, 2.127)	0.603
**N9**	12 (6.4)	20 (4.8)	1.548 (0.739, 3.243)	0.243
**N9a**	9 (4.8)	14 (3.3)	1.475 (0.627, 3.470)	0.371
**Y**	3 (1.6)	6 (1.4)	1.125 (0.278, 4.5470)	0.869
**R**	54 (28.9)	156 (37.1)	0.687 (0.473, 0.998)	0.048 *
**R11**	4 (2.1)	5 (1.2)	1.814 (0.482, 6.834)	0.372
**B**	19 (10.1)	74 (17.6)	0.529 (0.309, 0.904)	0.018 *
**B4**	13 (7.0)	52 (12.4)	0.529 (0.280, 0.997)	0.046 *
**B5**	6 (3.2)	19 (4.5)	0.717 (0.282, 1.8250)	0.484
**R9**	27 (14.4)	71 (16.9)	0.829 (0.513, 1.342)	0.446
**F**	21 (11.2)	62 (14.8)	0.730 (0.431, 1.239)	0.242
**F1**	13 (7.0)	38 (9.0)	0.751 (0.390, 1.446)	0.390
**F1a**	4 (2.1)	25 (6.0)	0.350 (0.12, 1.0)	0.045 *
**F1b**	6 (3.2)	8 (1.9)	1.707 (0.584, 4.991)	0.323
**F2**	8 (4.2)	17 (4.0)	1.059 (0.449, 2.500)	0.895
**F2a**	4 (2.1)	7 (1.67)	1.290 (0.373, 4.460)	0.687

*p* values were estimated by “chi-square test”;

(*) indicated statistical significant (*p* < 0.05);

OR indicates odds ratio; 95% CI, 95% confidence interval; values in ( ) are the percentage (%) of samples.

**Table 3.** Effect of mitochondrial DNA SNPs in patients with knee OA.

mtSNP	Patient (*n* = 187)	Control (*n* = 420)	*OR* (95% CI)	*p* value
**A10397G**	21 (11.2)	32 (7.6)	1.534 (0.859, 2.739)	0.146
**A10398G**	118 (63.1)	244 (58.1)	1.234 (0.865, 1.759)	0.246
**C10400T**	103 (55.1)	211 (50.2)	1.215 (0.859, 1.716)	0.270
**A16182C**	23 (12.3)	52 (12.4)	0.992 (0.588, 1.676)	0.987
**T16362C**	91 (48.7)	149 (35.5)	1.724 (1.215, 2.445)	0.002 *
**G16129A**	28 (15.0)	84 (20.0)	0.704 (0.441, 1.124)	0.140
**T16140C**	6 (3.2)	32 (7.6)	0.402 (0.165, 0.978)	0.038 *
**A16183C**	54 (28.9)	104 (24.8)	1.263 (0.859, 1.857)	0.234
**T16189C**	64 (34.2)	158 (37.6)	0.863 (0.601, 1.238)	0.423
**T16217C**	13 (7.0)	52 (12.4)	0.529 (0.280, 0.997)	0.046 *
**C16223T**	126 (67.4)	250 (59.5)	1.405 (0.977, 2.018)	0.066
**T16298C**	25 (13.4)	45 (10.7)	1.286 (0.763, 2.168)	0.344
**T16304C**	25 (13.4)	74 (17.6)	0.722 (0.442, 1.178)	0.191
**T16311C**	24 (12.8)	74 (17.6)	0.688 (0.419, 1.131)	0.139
**G16319A**	30 (16.0)	54 (12.9)	1.338 (0.828, 2.162)	0.233
**T146C**	26 (13.9)	64 (15.2)	0.898 (0.549, 1.470)	0.669
**C150T**	40 (21.3)	88 (21.0)	1.027 (0.674, 1.564)	0.903
**T152C**	59 (31.6)	103 (24.5)	1.419 (0.970, 2.075)	0.071
**249del**	34 (18.2)	95 (22.6)	0.760 (0.491, 1.176)	0.760
**T16172C**	16 (8.6)	49 (11.7)	0.708 (0.392, 1.281)	0.253

*p* values were estimated by “chi-square test”;

(*) indicated statistical significant (*p* < 0.05);

OR indicates odds ratio; 95% CI, 95% confidence interval; values in ( ) are the percentage (%) of samples.

**Table 4.** Effect of mitochondrial DNA haplogroup in OA development.

Haplogroup/SNPs	K/L = 2, 3 (*n* = 108)	K/L = 4 (*n* = 76)	*OR* (95% CI)	*p* value
**M**	57 (52.8)	45 (59.2)	1.299 (0.717, 2.353)	0.387
**D**	21 (19.4)	19 (25.0)	1.381 (0.683, 2.793)	0.368
**D4**	6 (5.6)	11 (14.5)	2.874 (1.014, 8.130)	0.04 *
**D4a**	0 (0)	4 (5.3)	2.500 (2.092, 2.994)	0.016 *
**D5**	14 (13.0)	7 (9.2)	0.681 (0.261, 1.776)	0.431
**D5a**	1 (0.9)	4 (5.3)	5.952 (0.651, 55.556)	0.075
**M8**	14 (13.0)	7 (9.2)	0.681 (0.261, 1.776)	0.431
**CZ**	8 (7.4)	6 (7.9)	1.072 (0.356, 3.226)	0.902
**M8a**	6 (5.6)	1 (1.3)	0.227 (0.027, 1.923)	0.139
**M7**	4 (3.7)	4 (5.3)	1.445 (0.350, 5.952)	0.610
**G**	1 (0.9)	7 (9.2)	10.870 (1.307, 90.909)	0.007 *
**M9**	3 (2.8)	1 (1.3)	0.467 (0.048, 4.566)	0.503
**M10**	4 (3.7)	1 (1.3)	0.347 (0.038, 3.165)	0.327
**N**	51 (47.2)	31 (40.8)	1.299 (0.717, 2.353)	0.387
**A**	12 (11.1)	6 (7.9)	0.686 (0.245, 1.916)	0.470
**N9**	11 (10.2)	1 (1.3)	0.118 (0.015, 0.931)	0.016 *
**N9a**	9 (8.3)	0 (0)	0.566 (0.497, 0.644)	0.01 *
**Y**	2 (1.9)	1 (1.3)	0.707 (0.063, 7.937)	0.777
**R**	32 (29.7)	22 (28.9)	0.968 (0.508, 1.845)	0.920
**R11**	2 (1.9)	2 (2.6)	1.433 (0.197, 10.417)	0.721
**B**	12 (11.1)	7 (9.2)	0.812 (0.304, 2.169)	0.677
**B4**	8 (7.4)	5 (6.6)	0.880 (0.277, 2.801)	0.829
**B5**	4 (3.7)	2 (2.6)	0.703 (0.125, 3.937)	0.687
**R9**	16 (14.8)	11 (14.5)	0.973 (0.424, 2.232)	0.949
**F**	12 (11.1)	9 (11.9)	1.074 (0.429, 2.695)	0.878
**F1**	6 (5.6)	7 (9.2)	1.724 (0.556, 5.348)	0.341
**F1a**	3 (2.8)	1 (1.3)	0.227 (0.023, 2.227)	0.166
**F1b**	4 (3.7)	2 (2.6)	0.703 (0.125, 3.937)	0.687
**F2**	6 (5.6)	2 (2.6)	0.460 (0.090, 2.342)	0.338
**F2a**	1 (0.9)	3 (3.9)	0.467 (0.048, 4.566)	0.503

*p* values were estimated by “chi-square test”;

(*) indicated statistical significant (*p* < 0.05);

OR indicates odds ratio; 95% CI, 95% confidence interval; K/L, Kellgren/Lawrence; values in ( ) are the percentage (%) of samples.

**Table 5.** Effect of mtSNPs in OA development.

mtSNP	K/L = 2, 3 (*n* = 108)	K/L = 4 (*n* = 76)	*OR* (95% CI)	*p* value
**A10397G**	14 (13.0)	7 (9.2)	0.681 (0.261, 1.776)	0.431
**A10398G**	68 (63.0)	49 (64.5)	1.067 (0.579, 1.965)	0.843
**C10400T**	57 (52.8)	45 (59.2)	1.299 (0.717, 2.353)	0.387
**A16182C**	12 (11.1)	11 (14.5)	1.353 (0.563, 0.563)	0.497
**T16362C**	46 (42.6)	42 (55.3)	1.664 (0.922, 3.012)	0.09
**G16129A**	15 (13.8)	13 (17.1)	1.279 (0.570, 2.874)	0.550
**T16140C**	5 (4.6)	1 (1.3)	0.275 (0.031, 2.398)	0.213
**A16183C**	33 (30.6)	21 (27.7)	0.868 (0.454, 1.658)	0.668
**T16189C**	38 (35.2)	26 (34.2)	0.958 (0.517, 1.776)	0.891
**T16217C**	8 (7.4)	5 (6.6)	0.880 (0.276, 2.801)	0.829
**C16223T**	72 (66.7)	51 (67.1)	1.020 (0.547, 1.905)	0.950
**T16298C**	18 (16.7)	7 (9.2)	0.507 (0.201, 1.282)	0.146
**T16304C**	12 (11.1)	13 (17.1)	1.650 (0.708, 3.846)	0.243
**T16311C**	13 (12.0)	12 (15.8)	1.370 (0.588, 3.195)	0.465
**G16319A**	18 (16.7)	10 (13.2)	0.758 (0.328, 1.748)	0.514
**T146C**	19 (17.6)	7 (9.2)	0.475 (0.189, 1.195)	0.108
**C150T**	29 (26.9)	12 (15.8)	0.511 (0.241, 1.08)	0.076
**T152C**	31 (28.8)	25 (32.9)	1.218 (0.646, 2.299)	0.543
**249del**	18 (16.7)	13 (17.1)	1.032 (0.472, 2.257)	0.938
**T16172C**	6 (5.6)	10 (13.2)	2.577 (0.894, 7.407)	0.072

*p* values were estimated by “chi-square test”; OR indicates odds ratio; 95% CI, 95% confidence interval; K/L, Kellgren/Lawrence; values in ( ) are the percentage (%) of samples.
